# Flexor Pollicis Brevis Muscle. Anatomical Study and Clinical Implications

**DOI:** 10.2174/1874325001711011321

**Published:** 2017-11-23

**Authors:** Edie Benedito Caetano, Yuri da Cunha Nakamichi, Renato Alves de Andrade, Maico Minoru Sawada, Mauricio Tadeu Nakasone, Luiz Angelo Vieira, Rodrigo Guerra Sabongi

**Affiliations:** Department of Orthopedics of the Pontifical Catholic University of São Paulo, Paulo, Brazil

**Keywords:** Median nerve, Ulnar nerve, Hand innervation, Cannieu-Riché, Thenar muscles innervation, Anatomical finds

## Abstract

**Introduction:**

This paper reports anatomical study of nature, incidence, innervation and clinical implications of Flexor Pollicis Brevis muscle (FPB).

**Material and Methods:**

The anatomical dissection of 60 limbs from 30 cadavers were performed in the Department of Anatomy of Medical School of Catholic University of São Paulo.

**Results:**

The superficial head of FPB has been innervated by the median nerve in 70% and in 30% it had double innervation. The deep head of FPB were absent in 14%, in 65%, occurred a double innervation. In 17.5% by deep branch of ulnar nerve and in 3.6% by recurrent branch of median nerve.

**Conclusion:**

The pattern of innervation more frequent in relationship to the flexor pollicis brevis muscle and should be considered as a normal pattern is that superficial head receives innervation of branches of median nerve and the deep head receives innervation of ulnar and median nerve.

## 1 INTRODUCTION

An important part of the evolutionary success of the human being is due to the fact that our thumb is capable of opposing the rest of the hand. Thanks to this feature, we are able to manipulate objects and create tools. The muscles that act on the opposing movement of the thumb are innervated by the median and ulnar nerves. The basic anatomy of the median and ulnar nerves in the upper limb, particularly in the hand is well described in textbooks [1-5]. A variety of clinical aspects observed in the isolated lesions of median and ulnar nerves, does not agree with the classic pattern of innervation of the Flexor Pollicis Brevis muscle (FPB) [6, 7]. The better knowledge of the anatomical variations of median and ulnar nerves helps to understand both anatomic variations and paradoxic complains of sensory and motor loss of patients. There are controversies in the classic treatises of the literature regarding superficial and deep head FPB muscle. This variety of synonyms for the same muscular portion makes interpretation difficult and causes some confusion regarding the innervation of the superficial and deep heads of FPB muscle. Cruveilhier 1 (1834) describes that FPB muscle is composed of two heads superficial and deep. Other classic authors have the same opinion [2, 3]. Wood Jones [8] considers that the deep head of the FPB of Cruveilhier^1^is part of the oblique head of the adductor pollicis muscle. Bischoff [9] considers the deep head of the FPB muscle to be the first palmar interosseous muscle. This disagreement persists to the present day. This controversy which still leads to misunderstandings, especially in regard to nerve supply, and leads to a confusion in the diagnosis and treatment of median and ulnar nerve injuries [7]. There is a wide discrepancy in FPB muscle innervation and based on different methods of investigation that have produced increased results, such a electromyography studies, anatomical studies, nerve block, and clinical examination were employed. Clinical and electromyography studies suggest that FPB muscles can receive dual innervation from median and ulnar nerves [10, 11]^.^ Similarly, the dual innervation of the superficial head of the FPB directly by the deep branch of the ulnar nerve is also a variation that can modify the conduction pathway [10]^.^ These variations allow aberrant exchange of axons between the median and ulnar nerves. They can change the composition of the nerves in the distal part of the upper limbs, thereby affecting the nerve supply in the hand [11]. The nerve communications between the median and ulnar nerves in the forearm [12-14] (Martin-Gruber anastomosis), and in the palm of the hand [15, 16] (Cannieu-Riché anastomosis) are of considerable importance in the anatomical variations innervation of the FPB muscle. We have undertaken an anatomic study to better understand the nature and incidence of anatomic variations the flexor pollicis brevis muscle innervation.

## 2 MATERIALS AND METHODS

Sixty hands of 30 fresh adult cadavers were dissected from 1983 to 2015. In all subjects, both hands were studied. Careful dissections were performed under high magnification (with a surgical microscope) to permit fine dissections. The ages ranged from 17 to 68 years, and the sex distribution was 26 males and 4 females that were available in the Department of Anatomy of the Medical School of the Catholic University of São Paulo. An initial pilot study consisting of the dissection of 4 hands from 2 fresh cadavers was performed to familiarize us with the regional anatomy of the palmar surface of the hand and is not included in this paper. The results were recorded by photography and drawings. These dissections were performed through a palmar carpal tunnel-type incision that extended distally along the palmar surface of the hand. The palmar skin, subcutaneous tissue and palmar fascia were removed. The median nerve was identified at the proximal edge of the transverse carpal ligament, the ligament was divided, and the branches were distally dissected to each thenar muscles. The ulnar nerve was also identified in the wrist proximal to the Guyon canal, and its deep motor branch was followed distally until its communication with the branches of the median nerve with the help of 2.5X magnifying glass. The dissection was then inspected under a microscope using 10- to 16-fold magnification. We investigate carefully the innervation of the two heads of FPB muscle. Schematic drawings of the pieces were created and systematically photographed.

## 3 RESULTS

**Fig. (1):**
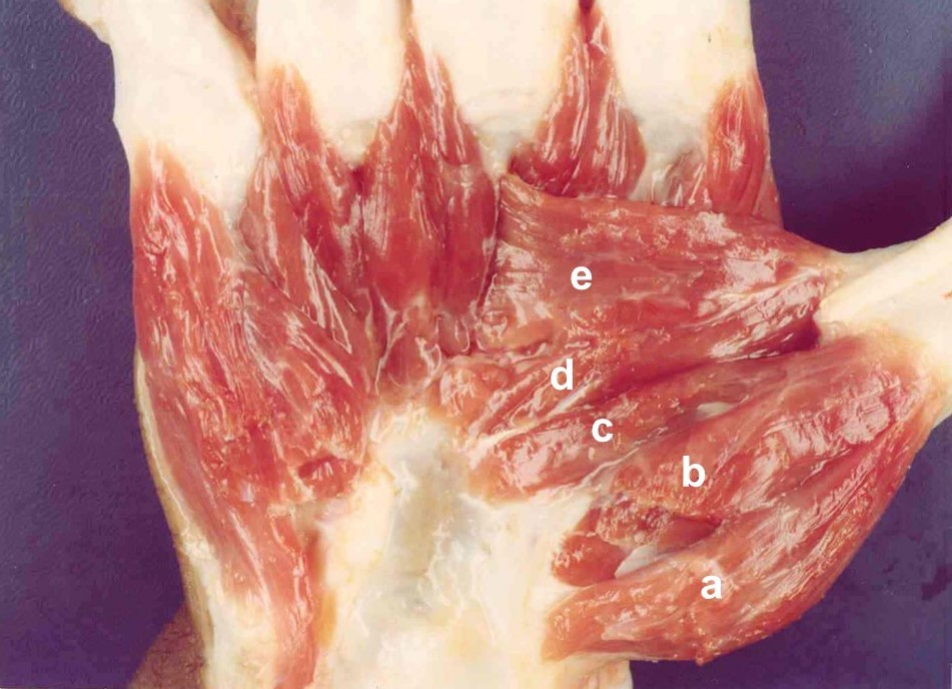
(a) Abductor pollicis brevis); (b) Superficial head of FPB; (c) Deep head of FPB; (d) Oblique head of Adductor pollicis; (e) Transverse head of adductor pollicis.

**Fig. (2):**
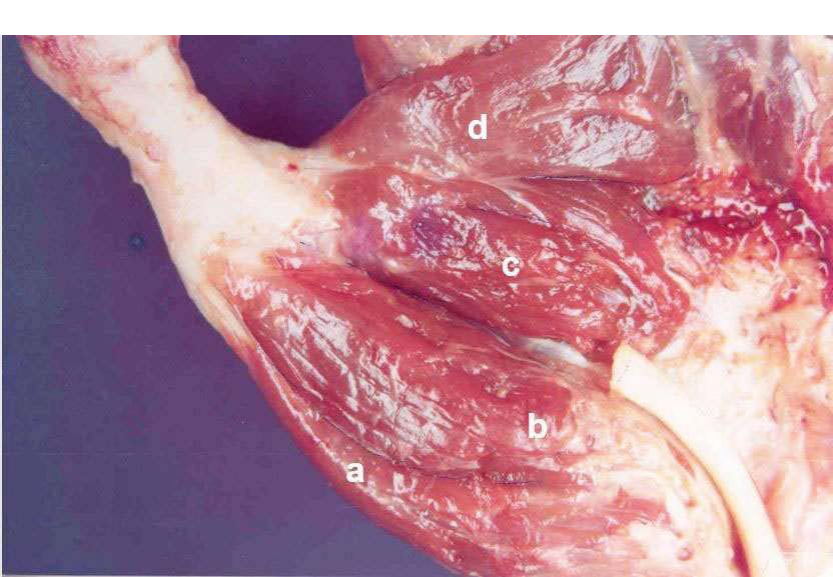
(a) Abductor pollicis brevis; (b) Superficial head of flexor pollicis brevis; (c) Oblique head of adductor pollicis; (d) Transverse head of adductor pollicis. Deep head of flexor pollicis brevis was absent.

**Fig. (3):**
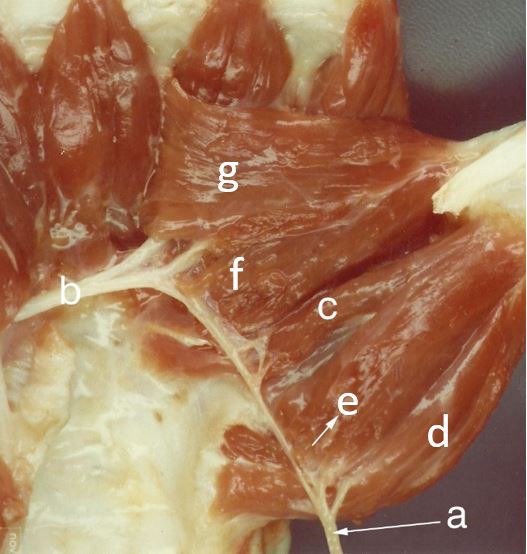
(a) Median nerve; (b) Ulnar nerve; (c) Deep head of FPB (double innervation); (d) Abductor pollicis brevis; (e) Superficial head of FPB exclusive by median nerve; (f) Oblique head of adductor pollicis; (g) Transverse head of adductor pollicis.

**Fig. (4):**
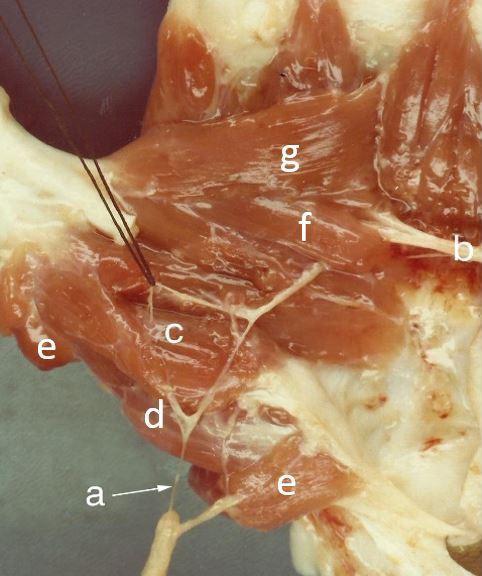
(a) Branch of median nerve; (b) Deep branch of ulnar nerve; (c) Superficial head of FPB (double innervation); (d) Oponens pollicis; (e) Adductor pollicis brevis; (f) Oblique head of adductor pollicis; (g) Transverse head of adductor pollicis.

The FPB had two heads; the superficial head arose from the crest of the trapezium and flexor retinaculum are present in sixty hands, and the deep head from the trapezoid and capitate bones and the palmar ligaments of the distal row of carpal bones. The deep head passed deep to flexor pollicis longus tendon and united with the superficial head on the radial sesamoid bone and the base of the proximal phalanx of the thumb (Fig. **1**). The deep head of FPB was absent in 9 hands (Fig. **2**). The superficial head of FPB muscle was innervated exclusively by the median nerve 42 hands (70%) (Fig. **3**), observations 1L, 1R, 2R, 3R, 4R, 6R, 9R, 10R, 11L, 11R, 12R, 12R, 13R, 14L 14R, 15L, 15R, 16R, 18R, 18L, 19L, 20L, 20R, 21L, 21R, 22L, 22R, 23L, 23R, 24L, 24R, 25L, 25R, 26L, 26R, 27R, 27L, 28R, 28L, 29R, 29L, 30L, with 16 bilateral and 10 unilateral occurrences (8 on the right side and only 2 on the left side), 37 males and 5 females, 26 whites and 16 non-whites. In the remaining 18 observations (30%), observations 2L, 3L, 5L, 5R, 6L, 7R, 7L, 8R, 8L, 9L, 10L, 13L, 16L, 17L, 17R, 19R, 30R, the superficial head FPB muscle received nervous fascicles from the median nerves and deep branch of ulnar nerve (Fig. **4**). This occurred 10 times in white individuals (9 males and 1 females) and 8 times in non-white individuals (all males), with 4 bilateral cases and 10 unilateral cases, that is, 8 on the left side and 2 on the right side. The exclusive innervation of the superficial head of flexor pollicis brevis muscle by the ulnar nerve was not verified in our preparations. In 25 preparations (41.6%), observations 1R, 2L, 6R, 8L, 9L, 10L, 10R, 11R, 12L, 15R, 15L, 16R, 17L, 18R, 18L, 19R, 19L, 21L, 22L, 22R, 25R, 26R, 28R, 28L, 30L, the fascicles of the median nerve to the superficial head of the FPB muscle were detached from its tenar motor branch, approaching this muscle by its palmar surface or by its medial border. In 18 observations, namely 2R, 3R, 4R, 5L, 5R, 7R, 7L, 9R, 11L, 13R, 14L, 16L,20R, 21R, 23L, 26L, 27R, 29L, the nervous supply of the superficial head of the FPB was made through nerve fascicles that originated directly from the median nerve at the level of its division at the distal edge of the flexor retinaculum. In 15 preparations, we found that the nervous fascicles to the superficial head of FPB originated from the collateral radial nerve of the thumb. In observation 3L, the superficial head of FPB muscle received nervous fascicles of the tenar motor branch of the median nerve and the radial collateral nerve of the thumb, at observation 13L it received fascicle from the tenar motor branch of the median and other fascicles directly from the median nerve.

**Fig. (5):**
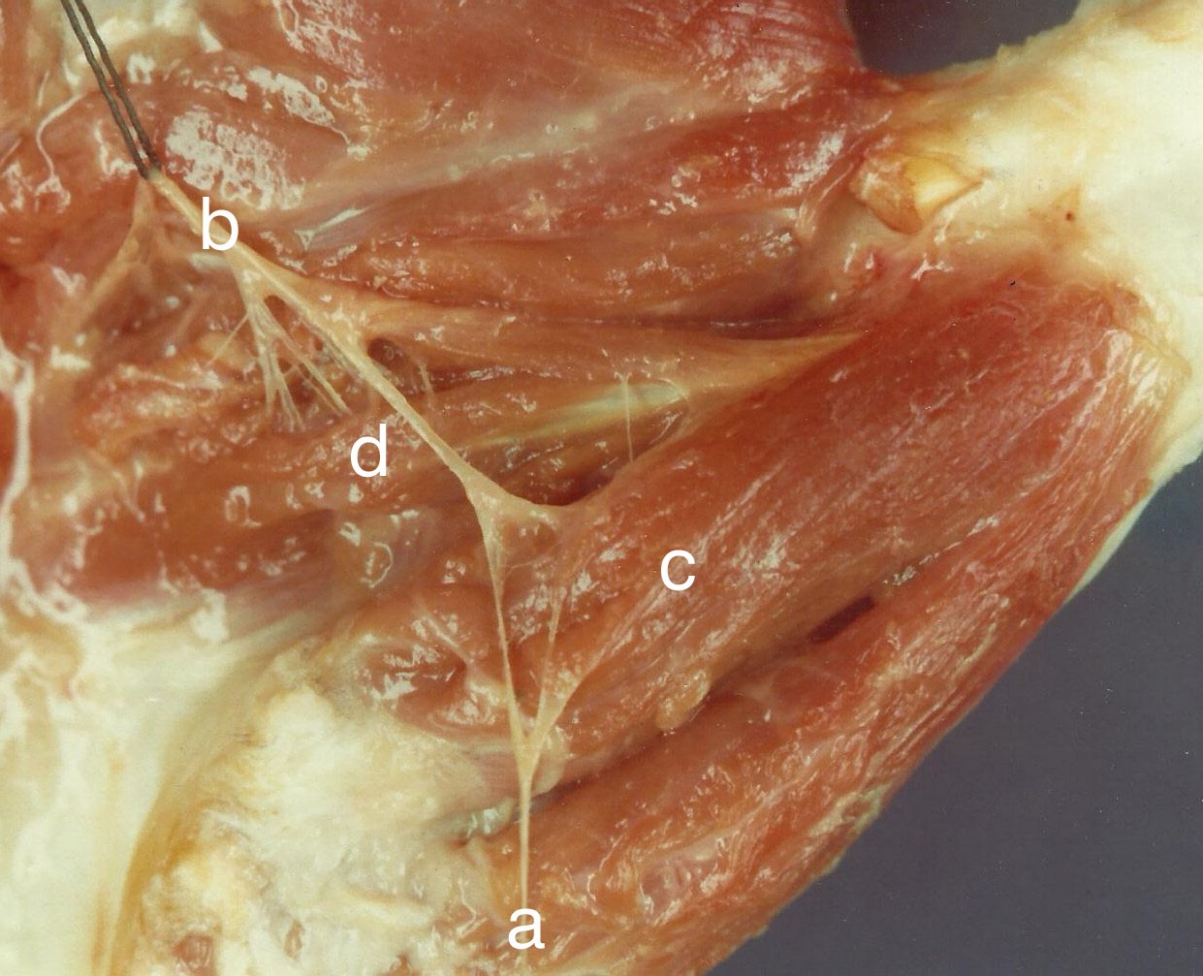
(a) Branch of median nerve; (b) Deep branch of ulnar nerve; (c) Superficial head of FPB (double innervation); (d) Deep head of FPB exclusive by ulnar nerve.

**Fig. (6):**
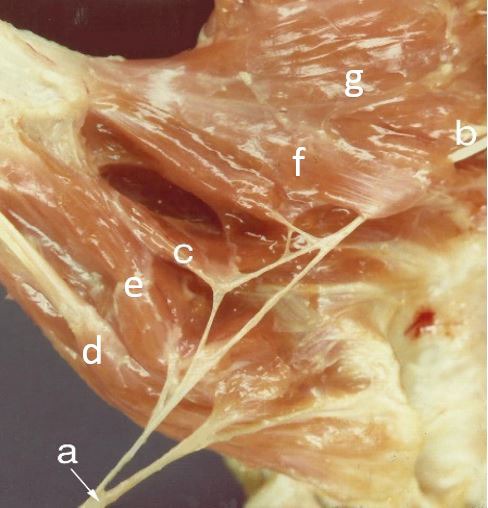
(a) Median nerve; (b) Deep branch of ulnar nerve; (c) Deep head of FPB (double innervation); (d) Abductor pollicis brevis; (e) Superficial head of FPB; (f) Oblique head of Adductor pollicis; (g) Transverse head of Adductor pollicis.

**Fig. (7):**
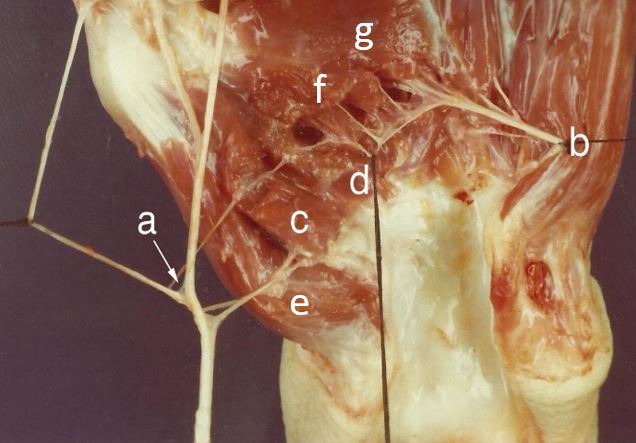
(a) Branch of median nerve; (b) Deep branch of ulnar nerve; (c) Superficial head of FPB and (d) Deep head of FPB (double innervation); (e) Abductor pollicis brevis; (f) Oblique head of Adductor pollicis; (g) Transverse head of Adductor pollicis.

The deep head of FPB muscle was not absent in 9 preparations (15%), observations 1R, 6R, 12L, 12R, 15R, 15L, 21L, 25L, 25R (Fig. **2**). Of the 51 specimens in which this muscle was present, only 14R (1.96%) preparation received innervation exclusive to the median nerve. In 10 preparations (19.6%), observations 2L, 3L, 4R, 7R, 8R, 9L, 9R, 16L, 17L, 21R the nerve supply was exclusive to the ulnar nerve (Fig. **5**). (Table **1**) This occurred in 6 white individuals (all males), and 4 non-whites (all males), with 2 bilateral occurrences and 6 unilateral cases (3 being only to the right and 3 only to the left). In the remaining 40 preparations (78.4%), observations 1L, 2R, 3R, 4L, 5L, 5R, 6L, 7L, 8L, 10L, 10R, 11L, 11R, 13L, 13R, 14L, 16R, 17 R,18L, 18R, 19L, 19R, 20L, 20R, 22L, 22R, 23L, 23R, 24L, 24R, 26L, 26R, 27L, 27R, 28L, 28R, 29L, 29R, 30L, 30D the deep head of FPB muscles were innervated by median and ulnar nerve (double innervation) (Fig. **6**), 21 males and 3 females, and in 16 non-white individuals (all males), of which 16 were bilateral and 8 unilateral (5 left and 3 right). The double innervation of both heads of FPB muscle by median and ulnar nerves was found in10 limbs (16,6%), observations 4L, 5L, 5R, 6E, 7E, 8E, 10E, 13L, 19R, 30R (Fig. **7**).

## 4 DISCUSSION

The variety of synonyms used to designate the two heads of FPB muscle, is responsible for the controversy surrounding this muscle. According to Day and Napier [7], the earliest description about FPB muscle in the literature appears to be that of Albinus [17], who described two heads, outer and inner. The outer corresponds to the deep head of FPB of Cruveilhier ^1^, and the inner corresponds to the oblique head of adductor pollicis of Wood Jones [8]. Bischoff [9] considers the deep head of the FPB muscle to be the first palmar interosseous muscle described by Henle [18]. Cruveilhier [1] described the muscle as having two heads, a superficial arising from flexor retinaculum and the adjacent portion of trapezium, and a deep head arising from the anterior carpal ligament, the trapezoid and the capitate. Brooks [6] outer head was equivalent to both the deep and the superficial heads of Cruveilhier [1]. This variety of synonyms for the same muscular portion makes interpretation difficult and causes some confusion regarding the innervation of the superficial and deep heads of FPB muscle. In this study our findings are similar to that described by Cruveilhier [1]: the FPB had two heads; the superficial head arose from the crest of the trapezium and flexor retinaculum are present in sixty hands, and the deep head from the trapezoid and capitate bones and the palmar ligaments of the distal row of carpal bones. The deep head passed deep to flexor pollicis longus tendon and united with the superficial head on the radial sesamoid bone and the base of the proximal phalanx of the thumb. The flexor pollicis brevis has been studied by Day and Napier [7] in a series of sixty-five hands from thirty-six male and female cadavers. The flexor pollicis brevis had two heads: the superficial head and the deep head. The deep head was present in sixty-two out of a total of sixty-five hands. In three cases the deep head was absent. In our study, we found two separate heads of flexor pollicis brevis muscle in 51 out of 60 dissected limbs, in 9. the deep head was absent.

The authors of classical anatomy treatises, such as Cruveilhier [1], Sunderland and Ray [2], Rouviere [3], kapan [4] and holinshead [5] consider the most accepted innervation pattern in relation to the flexor pollicis brevis is that superficial head of the FPB muscle receive innervation of the median nerve and deep head of the FPB muscle are innervated by the ulnar nerve. The variety of clinical pictures observed in the isolated lesions of the median and ulnar nerves is not in accordance with the classic innervation pattern of these muscles suggested by the authors of the anatomical treatises described above. The authors, Backhouse [19] and Brooks [6] reported that the ulnar nerve may occasionally innervate all the muscles of the hand. Marinacci [20] reports that using electrophysiological studies, he recorded the case of a patient who presented all the intrinsic muscles of the hand innervated by the median including the third and fourth lumbricis called this case as “All Median Hand”. Brandsma *et al.* [21] described the case of two patients with complete lesion of the median and ulnar nerves and had preserved the function of the flexor polliis brevis. Paraskevas [22] reports that during the dissection of an anatomical piece, he observed that the deep branch of the ulnar nerve besides innervating the adductor pollicis also supplied the two heads of flexor pollicis brevis muscle. Highet [10] reported a series of 20 median nerve lacerations and 25 ulnar nerve lacerations, analyzing the function of FPB. He found it to be present in 16 of 20 median nerve injuries and 24 of 25 ulnar nerve injuries. Forrest [11] has used electromyography and nerve stimulation to study variation in the motor supply about the thumb. His findings are similar to those of the Highet [10] showing that in 17 of 25 cases (68%). the FPB received dual innervation by the median and ulnar nerves. Rowntree [23] in a large series of World War II injuries, examined 102 median nerve lacerations and 124 ulnar nerve lacerations supplemented with electrical stimulation and local procaine nerve block and found that the FPB evidences double innervation in 15.5% of cases. Brooks [6] presented anatomic description of a double motor innervation of FPB in 19 of 31 hands (61%) and Forrest [11] electromyographic report showed dual innervation of the FPB in 17 of 25 cases (68%). According to our study, superficial head of flexor pollicis brevis has been innervated by the median nerve in 70% and in 30% it had double innervation. The deep head of FPB was absent in 14% and in 65%, a double innervation occurred. In 17.5% by deep branch of ulnar nerve and in 3.6% by recurrent branch of median nerve. We agree with opinion that the comparison of results obtained through anatomic dissections with studies through electrical stimulation should not be done, because according to the authors of eletromyografic studies, it is very difficult to separate the two heads of the FPB muscle. The authors Rowntree [23] in 15% and Sala [24] in 74% of the cases verified the double innervation of the FPB muscle, however the confrontation of results with these studies becomes impossible, since these authors used electromyographic methods for their execution and consider the FPB muscle as a single muscle, making no distinction between the superficial and deep heads. For example: Analyzing the authors opinion on the innervation of the opponens pollicis muscle, the double innervation of the median and ulnar nerves was recorded by Sala [24] in 32% of the cases, by Forrest [11] in 20% and Harness and Sequeles [25] in 77%. The explanation for our findings is so divergent as all these authors performed electrophysiological studies. When we compared the studies in which the authors used exactly the same technique and arrived at completely different results, we see that, according to Forrest [11], this fact occurred because the authors placed electrodes with different depths and that the highest percentage of Sala [24] occurred because they placed electrodes in contact with the muscle fibers of the FPB muscle. This fact demonstrates that the study through electrical stimulation is not as accurate as the study performed through anatomical dissections. Nerve communications between the median and ulnar nerves in the forearm (Martin-Gruber anastomosis) [12-14], and in the palm of the hand Cannieu-Riché's anastomosis [15, 16] are of considerable importance in the innervation of the intrinsic muscles of the hand.

## CONCLUSION

According to our studies with respect to the flexor pollicis brevis muscle, the superficial head receives innervation of the median nerve and the deep head receives innervation of the median and ulnar nerves (double innervation). The knowledge of anatomical variations in relation to hand intrinsic muscles innervation is relevant, especially when considering the physical examination, diagnosis, prognosis and surgical treatment. If these variations are not valued, errors and consequences will be unavoidable.

## Figures and Tables

**Table 1:** The nerve supply of flexor pollicis brevis in 60 hands of 30 cadavers.

Head	Median Nerve Supply Alone	Ulnar Nerve Supply Alone	Median and Ulnar Nerve Supply	Absent	Total Observations
Superficial	42 (70%)	--------------	18 (30%)	------------	60
Deep	1 (1,96%)	10 (19,6%)	40 (78,4%)	9 (15%)	60
